# Adaptive filter with Riemannian manifold constraint

**DOI:** 10.1038/s41598-023-36127-y

**Published:** 2023-06-02

**Authors:** Jose Mejia, Boris Mederos, Nelly Gordillo, Leticia Ortega

**Affiliations:** 1 Department of Electrical and Computer Engineering, Universidad Autónoma de Ciudad Juárez, 32310 Ciudad Juárez, Mexico; 2 Department of Physics and Mathematics, Universidad Autónoma de Ciudad Juárez, 32310 Ciudad Juárez, Mexico

**Keywords:** Electrical and electronic engineering, Applied mathematics

## Abstract

The adaptive filtering theory has been extensively developed, and most of the proposed algorithms work under the assumption of Euclidean space. However, in many applications, the data to be processed comes from a non-linear manifold. In this article, we propose an alternative adaptive filter that works on a manifold, thus generalizing the filtering task to non-Euclidean spaces. To this end, we generalized the least-mean-squared algorithm to work on a manifold using an exponential map. Our experiments showed that the proposed method outperforms other state-of-the-art algorithms in several filtering tasks.

## Introduction

Adaptive filtering has had several successful practical applications and plays an important role in signal processing. The fields in which adaptive filtering has been applied are system identification, channel equalization, biosignal noise cancellation, and acoustic echo cancellation, among others. The frequent use of adaptive filtering is necessary because signals are often contaminated by noise and unwanted artifacts, such as acoustic echo, power-line interference, and a mother’s heartbeat while trying to measure a fetal electrocardiogram. Until recently, most methods for adaptive filtering proposed changes to the objective function to optimize filter coefficients based on the assumption of Euclidean embedding. However, studies have shown that frequently data in signal processing possess latent non-Euclidean structures^1^, indicating that other spaces for filter design may provide more meaningful geometric representations and better signal-processing algorithms. This research expands the use of adaptive filtering beyond Euclidean domains by extending the least-mean-squared (LMS) algorithm to Riemannian manifolds. To accomplish this, we restrict the filter coefficients to exist within a specific manifold. As a result, the proposed optimization algorithm requires that the optimization steps occur on this particular structure.

The theory of adaptive filtering has been the subject of significant research, leading to the development of numerous algorithms. This paper seeks to extend the scope of adaptive filtering to non-Euclidean domains. Currently, most algorithms are designed for Euclidean domains and assume Gaussian noise. In practice, there can be other types of noise and interference that affect the performance of the algorithms. To address this issue, new algorithms have been proposed that modify the cost function by incorporating different schemes. For example, some works have proposed changing the error criteria in the optimization of the filter, such as the information theoretic criterion of minimum error entropy, which allows for better treatment of complex noise distributions^2,3^. Another concept related to Renyi’s entropy^4^, mixed correntropy, uses a convex linear combination kernel of two Gaussian functions. In this scheme, the maximum mixing correntropy criterion is used as the cost function; the result is a flexible and robust filter, that exhibits good performance in some scenarios. Furthermore, techniques based on compressive detection (CS) have also been incorporated, giving rise, for example, to zero attraction algorithms, in which a penalty is imposed that allows sparsity in the cost function. For example, Wang et al. (2018)^5^ introduced a bias-compensation vector to compensate for the bias resulting from an input with noise. For this, an $l_1$-norm penalty is used in the cost function that favors sparsity.

The LMS algorithm was designed to work with filter coefficients varying freely in ${\mathbb {R}}^n$, however, practical scenarios often involve filter coefficients subject to constraints imposed by physical characteristics or other influencing factors. As a result, the LMS algorithm is inadequate for determining the filter coefficients when they are limited to a specific subset of the Euclidean space ${\mathbb {R}}^n$. Instead, constrained optimization methods are employed^6^. Some examples of LMS with restrictions arise naturally in various applications such as array processing, spectral analysis and blind multiuser detection where the filters coefficients are subject to a set of linear equality constraints. To address this, the LMS algorithm with a linear equality constraint was proposed in^7,8^, achieving a better performance, while box constraints were utilized in^9,10^ where it was further extended to bounded norm constraints with $l_2, l_1, l_{\infty }$ norms. In^11,12^ an adaptive filter algorithm incorporating quadratic equality constraint was introduced. The work of^6^ introduced two distinct types of constraints, the bounded hypercube in ${\mathbb {R}}^n$ (box-constrains) and the bounded hypersphere which led to the development of a quadratically constrained algorithm. The work of^13^ extends the LMS method to incorporate filter coefficients constrains specified by general sets of constraints defined by convex functions. Particularly,^14^ demonstrates that their proposed constraint/regularization methods effectively ensure the filter parameters to satisfy the constraints, resulting in superior performance compared to the traditional LMS algorithm.

In the literature mentioned earlier, several commonly encountered sets of constraints can be understood as Riemannian manifolds. For example, the task of minimizing a general function $f:{\mathbb {R}}^n \rightarrow {\mathbb {R}}$ over the hypercube in ${\mathbb {R}}^n$ can be reformulated as a minimization problem on a manifold, as stated in^15^. Likewise, optimizing $f:{\mathbb {R}}^n \rightarrow {\mathbb {R}}$ subject to quadratic inequality constraints corresponds to optimization on the hypersphere, which constitutes a Riemannian manifold. Furthermore, the scenario involving linear constraints can be straightforwardly translated into an optimization problem on the linear manifold defined by the set of linear constraints. Therefore, in several cases, including the aforementioned ones, the LMS algorithm with constrains can be formulated as an optimization method on a manifold, thereby leveraging the advantages provided by such techniques^16^.

In this research, we explore the practical benefits of utilizing novel non-Euclidean spaces for filter design. We anticipate that the community will further advance this approach theoretically and uncover new applications for it. To our knowledge, the only similar work is from Bonnabel et al.^17^, which in the context of learning problems for classification and clustering, proposes a generalization of LMS to the particular case of the manifold of low-rank positive semidefinite matrices. Their proposed algorithm does not explicitly use the exponential map in the optimization process. In contrast, our research focuses on signal processing and expands the LMS algorithm to a broader context where the filter coefficients exist on a geodesically complete Riemannian manifold. To perform optimization on a manifold, we use the exponential map. Our study demonstrates that the algorithm converges in this new context.

The rest of the paper is organized as follows. In the Mathematical Preliminaries section, the main mathematical concepts from Riemannian geometry and the stochastic gradient descent used in this work are introduced. In the Methods section, we review the LMS and normalized least mean square (NLMS) algorithms and their geometric interpretations, and then generalize these results to other spaces. We then propose an algorithm for adaptive filtering on varieties based on LMS and the exponential map. In the Results section, a comparison of the performance of the proposed method against other algorithms for various tasks is presented. Finally, in the Conclusions section, a description of the results obtained is presented.

## Mathematical preliminaries

This section, presents the fundamentals of Riemannian geometry underlying the optimization theory over manifolds. Also, a brief introduction to the stochastic gradient on manifolds is presented. In this work, the manifolds $\mathscr {M}$ under consideration are submanifolds embedded in the Euclidean space ${{\mathbb {R}}}^n$ for some *n*. We start by giving some basics definition, which can be found in several books on Riemannian geometry^18–21^, among others.

### Definition 1

(*Tangent space*) Given $x \in {\mathscr {M}}$, the tangent plane at a point *x* is defined as:

$$\begin{aligned} T_x {\mathscr {M}} = \{c'(0): c: I\rightarrow {\mathscr {M}} \; \text {is smooth and} \; c(0) = x\}, \end{aligned}$$

where *I* is any open interval containing $t = 0$.

That is, $v \in T_x {\mathscr {M}}$ if and only if there exists a smooth curve on ${\mathscr {M}}$ passing through *x* with velocity *v*. It can be proved that $T_x {\mathscr {M}}$ is a lineal space of the same dimension as the manifold ${\mathscr {M}}$. Next, the concept of disjoint union of all the tangent spaces of the manifold is formalized.

### Definition 2

(*Tangent bundle*) The tangent bundle of a manifold ${\mathscr {M}}$ is denoted $T{\mathscr {M}}$ and is defined as:

$$\begin{aligned} T{\mathscr {M}} = \{ (x, v): x \in {\mathscr {M}} \; \text {and} \; v \in T_x {\mathscr {M}}\}. \end{aligned}$$

On the tangent space $T_x {\mathscr {M}}$ an inner product $\langle \cdot , \cdot \rangle _x : T_x {\mathscr {M}} \times T_x {\mathscr {M}} \rightarrow {\mathbb {R}}$ can be defined, it induces a norm $\Vert u\Vert _x = \sqrt{\langle u, u \rangle _x}$. When the metric $\langle \cdot , \cdot \rangle _x$ varies smoothly, it defines a Riemannian metric.

### Definition 3

(*Riemannian metric*) Given a smooth manifold ${\mathscr {M}}$, a Riemannian metric is a correspondence that associates points $x\in {\mathscr {M}}$ to inner products $\langle \cdot , \cdot \rangle _x$ that varies smoothly with *x*. In other words, for all smooth vector fields *X*, *Y* on ${\mathscr {M}}$ the function $s: {\mathscr {M}} \rightarrow {\mathbb {R}}$ defined as $s(x)= \langle X(x), Y(x) \rangle _x$ is smooth. A manifold with a Riemannian metric is called a Riemannian manifold.

In our context, where ${\mathscr {M}}$ is a submanifold embedded in the Euclidean space ${\mathbb {R}}^n$ for some *n*, we have that $\langle \cdot , \cdot \rangle _x$ is the inner product of ${\mathbb {R}}^n$.

Given a smooth function $f:{\mathscr {M}} \rightarrow {\mathscr {M}}'$ between two smooth manifolds ${\mathscr {M}}$ and ${\mathscr {M}}'$, the differential of *f* at a point $x \in {\mathscr {M}}$ is denoted as $df_x:T_x {\mathscr {M}} \rightarrow T_{f(x)} {\mathscr {M}}'$ and defined as $df_x(v) =(f \circ c)'(0)$, where $v\in T_x {\mathscr {M}}$ and $c:I\rightarrow {\mathscr {M}}$ is a curve that satisfies $c(0)=x$ and $c'(0)=v$. This allow to define the gradient vector field.

### Definition 4

(*Gradient*) Let $f: {\mathscr {M}} \rightarrow {\mathbb {R}}$ be a smooth function on a Riemannian manifold ${\mathscr {M}}$. The Riemannian gradient of *f* is the vector field $\nabla f$ on *M* that satisfies the following identity:

$$\begin{aligned} df_x(v) = \langle v, \nabla f(x) \rangle _x,\; \forall (x, v) \in T {\mathscr {M}}, \end{aligned}$$

where $\langle \cdot , \cdot \rangle _x$ is the Riemannian metric on ${\mathscr {M}}$.

Given a smooth function $f: {\mathscr {M}} \rightarrow {\mathbb {R}}$, the gradient $\nabla f$ is uniquely defined. Moreover, the gradient $\nabla f$ can be calculated from a smooth extension ${\bar{f}}:{\mathbb {R}}^n \rightarrow {\mathbb {R}}$ of *f* as

$$\begin{aligned} \nabla f(x) = \text {Proj}_x(\nabla {\bar{f}}(x)), \end{aligned}$$

where $\text {Proj}_x$ is the orthogonal projection from ${\mathbb {R}}^n$ to $T_x {\mathscr {M}}$.

Typically, the geodesic curves are defined in terms of the covariant derivative; here we give a definition based on the fact that the manifold is embedded in a Euclidean space, see Boumal^20^.

### Definition 5

(*Geodesics*) On a Riemannian manifold ${\mathscr {M}}$, a geodesic is a smooth curve $c:I \rightarrow {\mathscr {M}}$ such that its intrinsic acceleration $c''(t) = \text {Proj}_x \left( \frac{d^2c(t)}{dt^2} \right) = 0, \;\forall t \in I$.

Given $p\in {\mathscr {M}}$, consider $V \subset {\mathscr {M}}$ open set containing *p*. Let us define

$$\begin{aligned} {\mathscr {U}} = \{(q,v) \in T {\mathscr {M}}: q\in V, v\in T_q {\mathscr {M}}, \Vert v\Vert _q<\epsilon \} \end{aligned}$$

for some $\epsilon >0$. Then, the following Lemma from Do Carmo et al.^18^[Proposition 2.7, Page 64], holds

### Lemma 1

*Given* $p\in {\mathscr {M}}$, *there exist an open set* $V\subset {\mathscr {M}}$, $p\in V$, *numbers* $\epsilon >0$, $\delta > 0$ *such that* $\forall (q,v)\in {\mathscr {U}}$ *there exists a curve* $c_{q,v}:(-\delta , \delta )\rightarrow M$ *which is the unique geodesic satisfying* $c_{q,v}(0)=q$ *with velocity* $c_{q,v}'(0)=v$.

This Lemma guarantees the existence of a unique geodesic that passes by *p* with velocity *v* satisfying $\Vert v\Vert _p<\epsilon$. From this, the exponential map $\text {Exp}: {\mathscr {U}} \subset T {\mathscr {M}} \rightarrow {\mathscr {M}}$ can be defined as

$$\begin{aligned} \text {Exp}(q,v)=c_{q,v}(1),\; \forall (q,v) \in {\mathscr {U}}. \end{aligned}$$

It is common to consider the restriction of $\text {Exp}$ to the tangent plane in the following way, given a point $q \in {\mathscr {M}}$, the function $\text {Exp}_q:B(0,\epsilon ) \rightarrow {\mathscr {M}}$ is defined as

$$\begin{aligned} \text {Exp}_q(v) = \text {Exp}(q,v) \end{aligned}$$

where $B(0,\epsilon ) = \{ v \in T_q {\mathscr {M}} : \Vert v\Vert _q < \epsilon \}$ is an open ball with radius $\epsilon$ as in Lemma 1. $\text {Exp}_q(v)$ can be interpreted as a point of ${\mathscr {M}}$ obtained by walking along the geodesic for a time equal to one unit with velocity $\Vert v\Vert _q$.

The value of $\epsilon$ can be chosen such that $\text {Exp}_q:B(0,\epsilon ) \rightarrow {\mathscr {M}}$ is a diffeomorphism over its image, the supreme of the values of $\epsilon$ such that $\text {Exp}_q$ is still a diffeomorphism defines the injectivity radius. A formal definition is presented below

### Definition 6

(*Injectivity radius*) The injectivity radius of a Riemannian manifold ${\mathscr {M}}$ at *x* is denoted as $\text {inj}(x)$. It is defined as the supremum of values of $\epsilon >0$ such that the exponential map is a global diffeomorphism from $B(0,\epsilon ) \subset T_x {\mathscr {M}}$ over its image on ${\mathscr {M}}$.

The injectivity radius of ${\mathscr {M}}$, is defined as the minimum value of $\text {inj}(x)$, this is,

$$\begin{aligned} \text {inj}({\mathscr {M}})=\min _{x \in {\mathscr {M}}} \text {inj}(x). \end{aligned}$$

### Definition 7

(*Geodesically complete*) A Riemannian manifold ${\mathscr {M}}$ is geodesically complete if the maximal interval where the geodesics are defined is ${\mathbb {R}}$.

Observe that in a geodesically complete Riemannian manifold, the exponential maps are defined in the entire tangent plane. Some examples of geodesically completed manifolds are the sphere ${\mathbb {S}}^n$, the *n*-dimensional torus ${\mathbb {T}}^n$, and the hyperbolic space ${\mathbb {H}}^{n-1}$. In this work, we will restrict to geodesically complete manifolds.

Now we review the stochastic gradient descent algorithm (SGD) and its Riemannian version. Given a cost function

1 $$\begin{aligned} C(w)=E_{z}[f(z,w)], \end{aligned}$$

defined as the expected value of the loss function *f*(*z*, *w*) with respect to the variable *z*, the gradient of *C*(*w*) is expressed as

2 $$\begin{aligned} \nabla C(w)=E_{z}[h(z,w)] \end{aligned}$$

where

3 $$\begin{aligned} h(z,w) =\nabla _w f(z,w). \end{aligned}$$

To avoid the complexity of the function (2) in the minimization of *C*(*w*), the SGD algorithm starts from an initial value $w_1$ and interactively takes samples $z_k$ of the variable *z* and for each sample calculates a new value of *w* by the following iterative scheme

4 $$\begin{aligned} w_{k+1}=w_k - \rho _k h(z_k,w_k). \end{aligned}$$

The convergence of the SGD algorithm have been proved by Bottou in^22^. This method can be generalized to a Riemannian manifold ${\mathscr {M}}$ where the optimization problem is stated as

$$\begin{aligned} \min _{ w\in {\mathscr {M}}} C(w), \end{aligned}$$

under this context, the iterative scheme (4) is carried out by means of the exponential map $w_{k+1} = exp_{w_k}(- \rho _k h(z_k,w_k))$ which allows to move over the manifold. The algorithm is summarized below. The convergence of the SGD on a Riemannian manifold was first studied proved by^23^, and subsequently several works such as Zhang et al.^24^, Tripuraneni’s et al.^25^, among others have demonstrated the convergence of modifications of the above algorithm. The next Theorem is due to Bonnabel^23^ and guarantees convergence on quite general conditions on ${\mathscr {M}}$ and *f*.
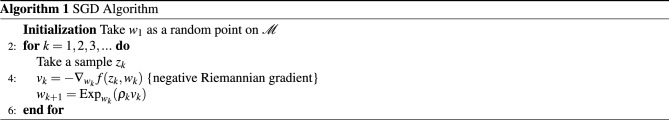


### Theorem 1

(Bonnabel) *Consider the SGD Algorithm 1 on a connected Riemannian manifold* ${\mathscr {M}}$ *with an injectivity radius uniformly bounded from below by* $I>0$. *Assume the sequence of step sizes* $\{\rho _k\}_{k=1}^{\infty }$ *satisfies the standard condition*.

5 $$\begin{aligned} \sum _{k=1}^{\infty } \rho _k^2 <\infty , \; \sum _{k=1}^{\infty } \rho _k =\infty . \end{aligned}$$

*Suppose that*:

1. *There exists a compact set* $K \subset {\mathscr {M}}$ *such that*$w_k \in K$ *for all* *k*.
2. *There exists a constant* $A > 0$ *such that* $\forall w \in K$ *and* $\forall z \in Z$ *we have* $h(z,w) \le A$, *where* *h*(*z*, *w*) *is the Riemannian gradient of the loss function* *f*(*z*, *w*).

*Then* $C(w_k)$ *converges a.s. and* $\nabla C(w_k) \rightarrow 0$ *a.s*.

## Methods

**Figure 1:**
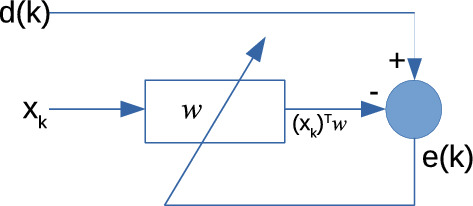
Adaptive filter diagram.

As shown in Fig. 1, the algorithms for adapting a filter to the desired conditions seek to find the filter coefficients $w=[w(1), w(2),...,w(n)]^T$ that minimize the error $e_k$ between the filter output and a desired signal $d_k$.

In this work, the implementing of an adaptive algorithm with filter coefficients embedded in a Riemannian manifold is proposed. In order to implement gradient descent, the exponential map was used, as in Sun et al. (2019)^26^. Next, we present the insights that led us to this viewpoint.

### The least-mean-square algorithm

We start by reviewing the LMS algorithm, which can be seen as an application of the SGD to the adaptive filter problem (Fig. 1). The target of the LMS algorithm is to minimize the average error

6 $$\begin{aligned} \text {MSE}(w)=E_{d,x} \left[ \frac{1}{2}(d-w^Tx)^2 \right] \end{aligned}$$

with respect to the filter coefficients *w*, where *x* is the input signal and *d* is the output signal. Moreover, it shall be considered that the output signal *d* is related with *x* by the equation $d={\tilde{w}}^Tx +\eta$ for some ${\tilde{w}} \in {\mathbb {R}}^n$, where $\eta$ is a zero mean random variable with variance $\sigma ^2$, which is independent of *x*. Note that the function (6) has the same form as Eq. (1) with loss function $f(d,x,w)=\frac{1}{2}(d-w^T x)^2$. Consequently, applying the SGD algorithm produces the update of the filter coefficients $w_k$ at iteration *k* as

7 $$\begin{aligned} w_{k+1}=w_k+\rho _k\, (d_k-w_k^Tx_k)x_k, \end{aligned}$$

where $\rho _k > 0$ is the step size, $x_k$ is the input or reference signal

8 $$\begin{aligned} x_k=[ x(k), x(k-1),\dots ,x(k-n+1)]^T, \end{aligned}$$

and $e_k = d_k-w_k^T x_k$ is the filter error at iteration *k*.

**Figure 2:**
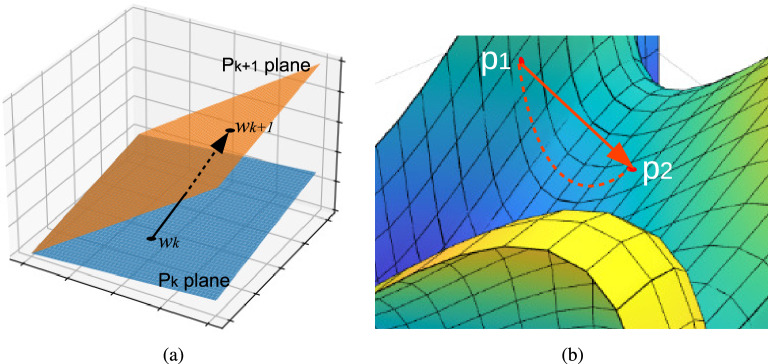
(**a**) The geometric interpretation of the NLMS algorithm involves projecting the present estimate, $w_k$, onto a hyperplane $p_{k+1}$ to obtain the next estimate, $w_{k+1}$. (**b**) When considering a path from point $p_1$ to point $p_2$ and employing Euclidean projection, a point on the path may exist outside the manifold, as indicated by the solid red line. However, this occurrence is absent when utilizing a geodesic curve, depicted as a dashed red line.

An approach to eliminating dependency in the “volume” of the signal, known as NLMS, suggests using the update Eq. (9) instead of Eq. (7)

9 $$\begin{aligned} w_{k+1}=w_k+\rho _k e_k\frac{x_k}{||x_k||_2^2}, \end{aligned}$$

where $\Vert \cdot \Vert _2$ is the Euclidean norm. The NLMS algorithm can be interpreted from a geometric viewpoint as projecting the current estimate $w_k$ on a hyperplane $p_{k+1}$ to find the next estimate $w_{k+1}$^27^(see Fig. 2a). The hyperplane $p_{k+1}$ is defined as

10 $$\begin{aligned} p_{k+1}=\{ w\in {\mathbb {R}}^n: d_k-w^T x_k=0 \}, \end{aligned}$$

which corresponds to the plane containing all points *w*, which make the error equal to zero. This process of finding a new plane and projecting onto it is performed at each iteration.

Here, we propose to view the iterative process as a whole, and instead of having a sequence of planes, we now have a manifold where each hyperplane is actually a tangent plane at a point on the manifold. Thus, instead of projecting into planes, what we need is a geodesic on the manifold in the direction of the error. This is illustrated in Fig. 2b, where, for a path from $p_1$ to $p_2$ (continuous line), a point in the path can be outside the manifold, not occurring for a geodesic curve (dashed curve).

The proposed LMS implementation is similar to the classic LMS algorithm (Eq. 7). However, we assume that the filter coefficients *w* are constrained to a smooth Riemannian manifold ${\mathscr {M}}$, which generalize other types of restrictions imposed in previous works^8–10^. Therefore, in this context, the LMS algorithm aims to solve the following optimization problem:

11 $$\begin{aligned} \min _{w \in {\mathscr {M}}}\text {MSE}(w), \end{aligned}$$

where the Riemannian manifold ${\mathscr {M}}$ embedded in the Euclidean space ${\mathbb {R}}^n$ for some *n*. which is endowed with a Riemannian metric which is the Euclidean inner product inherited from the ambient space ${\mathbb {R}}^n$.

As in the Euclidean *LMS*, the signal *d* satisfies that $d={\tilde{w}}^Tx+\eta$, where ${\tilde{w}}\in {\mathscr {M}}$ and $\eta$ is a random variable independent of *x* with zero means and variance $\sigma ^2$. The calculation of the filter output is performed as usually as a convolution of the FIR structure $w_k$ with the input $x_k$. To minimize $\text {MSE}(w)$ (Eq. 6) with $w \in {\mathscr {M}}$, the proposed LMS algorithm starts at an initial point $w_1 \in {\mathscr {M}}$ and progressively produces a sequence of filter values $\{w_k\}_{k=1}^{\infty }\subset {\mathscr {M}}$ in the same fashion as the SGD algorithm on manifold 1. Given a $w_k\in {\mathscr {M}}$ and a sample point $(d_k,x_k)$, the method computes the negative Euclidean gradient of $f(d_k,x_k,w) = \frac{1}{2}(d_k-w^Tx_k)^2$ as

12 $$\begin{aligned} h(d_k,x_k,w_k) = (d_k-w^Tx_k)x_k \end{aligned}$$

and the Riemannian gradient as the projection of Eq. (12) onto the tangent plane $T_{w_k} {\mathscr {M}}$

13 $$\begin{aligned} v_k = \text {Proj}_{w_k} \left( h(d_k,x_k,w_k) \right) . \end{aligned}$$

Then, the next point $w_{k+1}$ is obtained by moving $\rho _k>0$ units along the geodesic $\gamma _{w_k}(t)=\text {Exp}_{w_k}(tv_k)$ with velocity $v_k$, i.e.,

14 $$\begin{aligned} w_{k+1}=\text {Exp}_{w_k}(\rho _k v_k). \end{aligned}$$

This algorithm is summarized below
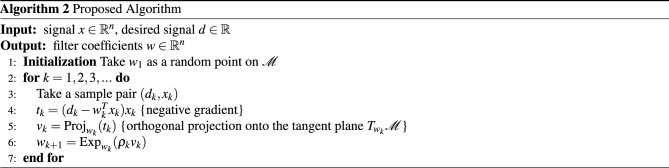


We implemented the proposed algorithm for two manifolds, the hypersphere ${\mathbb {S}}^{n-1}$ and the hyperbolic $n-1$-space ${\mathbb {H}}^{n-1}$ embedded in ${\mathbb {R}}^{n}$. Now, we present the specific expressions of the projection operators and exponential maps.

The hypersphere is defined as ${\mathbb {S}}^{n-1} =\{w\in {\mathbb {R}}^{n} : \langle w,w \rangle =1\}$ with Riemannian metric the Euclidean inner product inhered from ${\mathbb {R}}^{n}$. Given $w \in {\mathbb {S}}^{n-1}$, the tangent plane at *w* is $T_w{\mathbb {S}}^{n-1} = \{v\in {\mathbb {R}}^{n} : \langle v,w \rangle =0\}$, the orthogonal projection of ${\mathbb {R}}^{n}$ onto $T_w{\mathbb {S}}^{n-1}$ is

15 $$\begin{aligned} \text {Proj}_w(x)=(I-w w^T)x, \end{aligned}$$

and the exponential map is

16 $$\begin{aligned} \text {Exp}_{w}(v) = \cos (\Vert v\Vert )w +\sin (\Vert v\Vert )\frac{v}{\Vert v\Vert }. \end{aligned}$$

that can be founded in Boumal^20^.

The hyperbolic space is defined as ${\mathbb {H}}^{n}=\{y=(y_0, y_1, \dots , y_n)\in {\mathbb {R}}^{n+1}:\langle y,y \rangle _{{\mathbb {H}}^{n}} = -1, y_0>0\}$ with Riemannian metric $\langle u,v \rangle _{{\mathbb {H}}^{n}} =u^T J v$ known as the Minkowski inner product, where $J=\text{ diag }(-1,1,\dots ,1)$. The projection onto the tangent plane $T_{w_k} {\mathbb {H}}^{n}$ is given by

17 $$\begin{aligned} \text {Proj}_w(x)=x+\langle x,w \rangle _{{\mathbb {H}}^{n}} w \end{aligned}$$

The Riemannian gradient of a smooth function $f:{\mathbb {H}}^{n} \rightarrow {\mathbb {R}}$ and the exponential map can be founded in Boumal^20^[Proposition 7.7. Page 175]. Given an extension ${\bar{f}}$ of *f* to ${\mathbb {R}}^{n+1}$ the gradient of *f* can be calculated as

18 $$\begin{aligned} \text {Proj}_{w}(J\nabla {\bar{f}}(w)), \end{aligned}$$

and

19 $$\begin{aligned} \text {Exp}_{w}(v) = \cosh (\Vert v\Vert )w +\sinh (\Vert v\Vert )\frac{v}{\Vert v\Vert }. \end{aligned}$$

respectively.

To prove the convergence of algorithm 2, it is tempting to apply Theorem 1; however, this cannot be applied directly since the condition that the gradient $\text {Proj}_w\left( h(x,d,w) \right)$ is uniformly bounded for every pair (*d*, *x*) is not satisfied. However, following the ideas of the proof of Theorem 1 in Bonnabel^23^ the convergence of the proposed method can be proved with quite a general conditions of the random variable *x* as shown in the following Theorem.

#### Lemma 2

*Let* ${\mathscr {M}}$ *be a geodesically complete manifold embedded in* ${\mathbb {R}}^n$ *with Riemannian metric the traditional euclidean inner product inhered from* ${\mathbb {R}}^n$. *Let* $\text {MSE}: {\mathscr {M}} \rightarrow {\mathbb {R}}$ *as in Eq. * (6). *Assume that* $M=E_x\left[ \Vert x\Vert ^2xx^T \right]$ *and* $E_x\left[ \Vert x\Vert ^2 \right]$ *exist and that* *M* *is a strictly positive defined matrix*. *Consider that the Algorithm 2 is applied to* $\text {MSE}(w)$ *with the sequence of step sizes* $\{\rho _k\}_{k=1}^{\infty }$ *satisfying the standard condition* (Eq. 5) *and also assume that there exists a compact subset* $K \subset {\mathscr {M}}$ *such that* $w_k \in K$ *for all* *k*. *Then* $MSE(w_k)$ *converges a.s. and* $\nabla MSE(w_k) \rightarrow 0$ *a.s*.

#### Proof 1

A well-known fact is that for any *v* in a linear space *E* with inner product, the norm of the orthogonal projection $\text {Proj}_V(v)$ onto a linear subspace $V\subset E$ is smaller than the norm of *v*, this is

20 $$\begin{aligned} \Vert \text {Proj}_V(v)\Vert \le \Vert v\Vert . \end{aligned}$$

Therefore, defining ${{\tilde{h}}}(x,d,w)=\text {Proj}_w(h(x,d,w))$, we obtain

21 $$\begin{aligned} \Vert {{\tilde{h}}}(x,d,w)\Vert _w^2 \le \Vert h(x,d,w)\Vert ^2. \end{aligned}$$

Consequently

22 $$\begin{aligned} \Vert {{\tilde{h}}}(x,d,w)\Vert _w^2 \le \Vert h(x,d,w)\Vert ^2 = \Vert (d-w^Tx)x\Vert ^2=(d-w^Tx)^2\Vert x\Vert ^2. \end{aligned}$$

Using that $d={\tilde{w}}^Tx+\eta$, where ${\tilde{w}} \in {\mathscr {M}}$, we get

23 $$\begin{aligned} \Vert h(x,d,w)\Vert ^2 = \eta ^2 \Vert x\Vert ^2 + \Vert x\Vert ^2 \eta ({\tilde{w}}-w)^Tx + \Vert x\Vert ^2({\tilde{w}} - w)^T xx^T ({\tilde{w}} - w) \end{aligned}$$

Therefore, by the independence of $\eta$ and *x* and that $E_{\eta }\left[ \eta \right] =0$, then the following inequality holds

$$\begin{aligned} E_{d,x} \left[ \Vert {{\tilde{h}}}(x,d,w)\Vert ^2_w \right]\le & {} \,E_{\eta ,x}\left[ \eta ^2 \Vert x\Vert ^2 + \Vert x\Vert ^2 \eta ({\tilde{w}}-w)^Tx + \Vert x\Vert ^2({\tilde{w}} - w)^T xx^T ({\tilde{w}} - w) \right] \\= & {} \,E_{\eta } \left[ \eta ^2 \right] E_x \left[ \Vert x\Vert ^2 \right] + ({\tilde{w}} - w)^T E_{x} \left[ \Vert x\Vert ^2xx^T \right] ({\tilde{w}} - w) \\= & {} \,\sigma ^2 E_x \left[ \Vert x\Vert ^2 \right] + ({\tilde{w}} - w)^T E_{x} \left[ \Vert x\Vert ^2xx^T \right] ({\tilde{w}} - w). \end{aligned}$$

Then

24 $$\begin{aligned} E_{d,x}\left[ \Vert {{\tilde{h}}}(x,d,w)\Vert ^2_w \right] \le \sigma ^2 E_x \left[ \Vert x\Vert ^2 \right] + \lambda _* \Vert {\tilde{w}}-w\Vert ^2 \end{aligned}$$

where $\lambda _*>0$ is the greatest eigenvalue of $E_{x} \left[ \Vert x\Vert ^2xx^T \right]$. Since $w\in K$ and *K* is a compact set, then there exist some positive constant $C'>$ such that $\Vert {\tilde{w}}-w\Vert ^2 \le C', \forall w \in K$. This entails that

25 $$\begin{aligned} E_{d,x}\left[ \Vert {{\tilde{h}}}(x,d,w)\Vert ^2_w \right] \le \sigma ^2 E_x \left[ \Vert x\Vert ^2 \right] + \lambda _*C=C',\; \forall w \in K. \end{aligned}$$

Since ${\mathscr {M}}$ is geodesically complete, the exponential map $\text {Exp}_{w_k}(tv)$ is well-defined for all $t\in {\mathbb {R}}$ and $v \in T_{w_k} {\mathscr {M}}$, then, applying Taylor formula argument the same inequality (5) in^23^[Page 2219] with $w_{k+1} =\text {Exp}_{w_k}(-\rho _k {{\tilde{h}}}(x_k,d_k,w_k))$ is obtained

26 $$\begin{aligned} \text {MSE}(w_{k+1})- \text {MSE}(w_k) \le -\rho _k \left\langle {{\tilde{h}}}(x_k,d_k,w_k), \nabla MSE(w_k) \right\rangle _{w_k} + \rho _k^2 K \langle {{\tilde{h}}}(x_k,d_k,w_k), {{\tilde{h}}}(x_k,d_k,w_k)\rangle _{w_k}, \end{aligned}$$

where $\nabla MSE(w_k)$ is the Riemannian gradient of $MSE(w_k)$ as in the Definition 4 and $K>0$ is an upper bound of the eigenvalues of Hessian of the MSE function. Taking the expected value to both sides of Eq. (26) with respect to the sigma-algebra ${\mathscr {F}}_k=\{x_1, \eta _1,...,x_{k-1}, \eta _{k-1}\}$ and following the same arguments as in the proof of Theorem 1^23^, we get

27 $$\begin{aligned} E(\text {MSE}(w_{k+1})- \text {MSE}(w_k)|{\mathscr {F}}_k ) \le -\rho _k \Vert \nabla MSE(w_k)\Vert ^2_{w_k} + \rho _k^2 K E_{d_k,x_k}\left[ \Vert {{\tilde{h}}}(x_k,d_k,w_k)\Vert ^2_{w_k} \right] . \end{aligned}$$

Applying inequality (Eq. 25) to the last term of the previous inequality we obtain

28 $$\begin{aligned} E(\text {MSE}(w_{k+1})- \text {MSE}(w_k)|{\mathscr {F}}_k ) \le -\rho _k \Vert \nabla MSE(w_k)\Vert ^2_{w_k} + \rho _k^2 C'', \end{aligned}$$

where $C''=KC'$. The rest of the proof follows exactly the same as in the proof of Theorem 1 in^23^. Therefore, it is concluded that $MSE(w_k)$ converges a.s. and $\nabla MSE(w_k) \rightarrow 0$ a.s. $\square$

We remark that for a compact geodesically complete Riemannian manifold the compact set *K* in Lemma 2 can be considered as the whole manifold. The Hyperbolic space ${\mathbb {H}}^{n}$ is a geodesically complete manifold embedded in the euclidean space ${\mathbb {R}}^{n+1}$ but it does not inhered the inner product of ${\mathbb {R}}^{n+1}$ as its Riemannian metric, therefore, the Lemma 2 can not be applied directly. In Lemma 3, we provide a proof of the convergence of LMS algorithm when the filter coefficients are constrained to the Hyperbolic space ${\mathbb {H}}^{n}$. We recall that the Hyperbolic space is defined as

29 $$\begin{aligned} {\mathbb {H}}^{n}=\{y=(y_0, y_1, y_2, \dots , y_n)\in {\mathbb {R}}^{n+1}:\langle y,y \rangle _{{\mathbb {H}}^{n}} = -1, y_1>0\}, \end{aligned}$$

and the Minkowski inner product is

30 $$\begin{aligned} \langle u,v \rangle _{{\mathbb {H}}^{n}} =u^T J v, \end{aligned}$$

where $J=\text{ diag }(-1,1,...,1)$. For $u=(u_0,u_1,...,u_n) \in {\mathbb {H}}^{n}$, the following inequality holds

31 $$\begin{aligned} \Vert u\Vert _{{\mathbb {H}}^{n}}^2 =\langle u,u \rangle _{{\mathbb {H}}^{n}} =u_1^2+\dots +u_n^2 - u_0^2\le \Vert u\Vert ^2, \end{aligned}$$

where $\Vert u\Vert$ is the euclidean norm. The following Lemma guarantees the convergence of the LMS algorithm on the *n*-dimensional Hyperbolic space

#### Lemma 3

*Let* ${\mathbb {H}}^{n}$ *be the* *n*-*dimensional Hyperbolic space. Let* $\text {MSE}: {\mathbb {H}}^{n} \rightarrow {\mathbb {R}}$ *as in Eq. * (6). *Assume that* $M=E_x\left[ \Vert x\Vert ^2xx^T \right]$ *and* $E_x\left[ \Vert x\Vert ^2 \right]$ *exist and that* *M* *is a strictly positive defined matrix. Consider that the Algorithm 2 is applied to* $\text {MSE}(w)$ *with the sequence of step sizes* $\{\rho _k\}_{k=1}^{\infty }$ *satisfying the standard condition* (5) *and also assume that there exists a compact subset* $K \subset {\mathbb {H}}^{n}$ *such that* $w_k \in K$ *for all* *k*. *Then* $MSE(w_k)$ *converges a.s. and* $\nabla MSE(w_k) \rightarrow 0$ *a.s*.

#### Proof 2

Let us start by proving that given $w \in {\mathbb {H}}^{n}$, the projection satisfies

$$\begin{aligned} \Vert \text {Proj}_w (z)\Vert _{{\mathbb {H}}^{n}}^2 \le \Vert z\Vert ^2(1+4\Vert w\Vert ^2) \end{aligned}$$

with $\Vert z\Vert$ the euclidean norm.

By Eq. (17) we have $z' = \text {Proj}_w (z)=z+\langle w,z \rangle _{{\mathbb {H}}^{n}} w$, then

$$\begin{aligned} \langle z',z' \rangle _{{\mathbb {H}}^{n}}= & {} \langle z,z \rangle _{{\mathbb {H}}^{n}} + 2 \langle z,w \rangle _{{\mathbb {H}}^{n}}^2 + \langle z,w \rangle _{{\mathbb {H}}^{n}}^2 \langle w,w \rangle _{{\mathbb {H}}^{n}}\\= & {} \langle z,z \rangle _{{\mathbb {H}}^{n}} + \langle z,w \rangle _{{\mathbb {H}}^{n}}^2. \end{aligned}$$

Using the fact that $\langle z,z \rangle _{{\mathbb {H}}^{n}}\le \Vert z\Vert ^2$ (corresponding to inequality (Eq. 31)) we obtain that

32 $$\begin{aligned} \langle z',z' \rangle _{{\mathbb {H}}^{n}}\le & {} \,\Vert z\Vert ^2 + \langle z,w \rangle _{{\mathbb {H}}^{n}}^2 \nonumber \\= & {} \,\Vert z\Vert ^2 + (-z_0w_0 + z_1 w_1 + \dots +z_nw_n)^2 \nonumber \\= & {} \,\Vert z\Vert ^2 + (z_0w_0)^2 -2(z_0w_0)(z_1 w_1 + \cdots +z_nw_n) + (z_1 w_1 + \cdots +z_nw_n)^2. \end{aligned}$$

Applying $2ab \le a^2 + b^2$ and the Cauchy–Schwartz inequality to Eq. (32) we get

33 $$\begin{aligned} \langle z',z' \rangle _{{\mathbb {H}}^{n}}\le & {} \Vert z\Vert ^2 + 2(z_0w_0)^2 + 2(z_1 w_1 + \dots +z_nw_n)^2 \end{aligned}$$

34 $$\begin{aligned}\le & {} \Vert z\Vert ^2 + 2\Vert z\Vert ^2\Vert w\Vert ^2 + 2\Vert z\Vert ^2\Vert w\Vert ^2 = \Vert z\Vert ^2(1+4\Vert w\Vert ). \end{aligned}$$

Therefore, taking $z=h(x,d,w)$ and $z'=\text {Proj}_w(h(x,d,w))$ it is obtained

35 $$\begin{aligned} {\Vert {{\tilde{h}}}(x,d,w)\Vert ^2_{{\mathbb {H}}^{n}}}_w \le \Vert h(x,d,w)\Vert ^2(1+4\Vert w\Vert ^2). \end{aligned}$$

Using the Eq. (23)

36 $$\begin{aligned} \Vert h(x,d,w)\Vert ^2 = \eta ^2 \Vert x\Vert ^2 + \Vert x\Vert ^2 \eta ({\tilde{w}}-w)^Tx + \Vert x\Vert ^2({\tilde{w}} - w)^T xx^T ({\tilde{w}} - w) \end{aligned}$$

and taking expected value

37 $$\begin{aligned} E_{d,x} \left[ \Vert h(x,d,w)\Vert ^2 \right] = \sigma ^2 E_x \left[ \Vert x\Vert ^2 \right] + ({\tilde{w}} - w)^T E_{x} \left[ \Vert x\Vert ^2xx^T \right] ({\tilde{w}} - w). \end{aligned}$$

Then, taking the expected values of Eq. (35) and sustituting Eq. (37) we have

38 $$\begin{aligned} E_{d,x} \left[ {\Vert {{\tilde{h}}}(x,d,w)\Vert ^2_{{\mathbb {H}}^{n}}}_w \right] \le \left[ \sigma ^2 E_x \left[ \Vert x\Vert ^2 \right] + ({\tilde{w}} - w)^T E_{x} \left[ \Vert x\Vert ^2xx^T \right] ({\tilde{w}} - w) \right] (1+4\Vert w\Vert ^2). \end{aligned}$$

This inequality allows the bound $E_{d,x} \left[ {\Vert {{\tilde{h}}}(x,d,w)\Vert ^2_{{\mathbb {H}}^{n}}}_w \right] \le C$ as $w \in K$. From this upper bound of $E_{d,x} \left[ {\Vert {{\tilde{h}}}(x,d,w)\Vert ^2_{{\mathbb {H}}^{n}}}_w \right]$, we can proceed following the steps outlined in Lemma 2 and the Theorem 1 in^23^.$\square$

## Results

In this section, the results of the experiments are presented. The proposed algorithm was implemented using the *geostats* library^28^. For the comparison methods, the following methods were used: the LMS^29^, the kernel LMS (Kernel)^30^, the bias compensated zero-attracting normalized least mean square adaptive filter (BCZA) of^5^, and the normalized LMS adaptive filter with a variable regularization factor (NNLMS) of^31^. In all the experiments, a simple grid search to establish the parameters of the algorithms for each task was used. In all cases, the assumed manifold had a dimension equal to the filter order. The learning curves averaged over 100 realizations.

As a preliminary experiment, we introduce the identification of two systems whose models possess a spherical and hyperbolic manifold structure, respectively. The systems are represented using Finite Impulse Response (FIR) filters, with their coefficients derived from the corresponding manifold. The size of each filter is six dimensions. The signals were generated by convolving with a square wave of random and varying periods. Subsequently, the different methods were evaluated, and their respective learning error curves were plotted.

**Figure 3:**
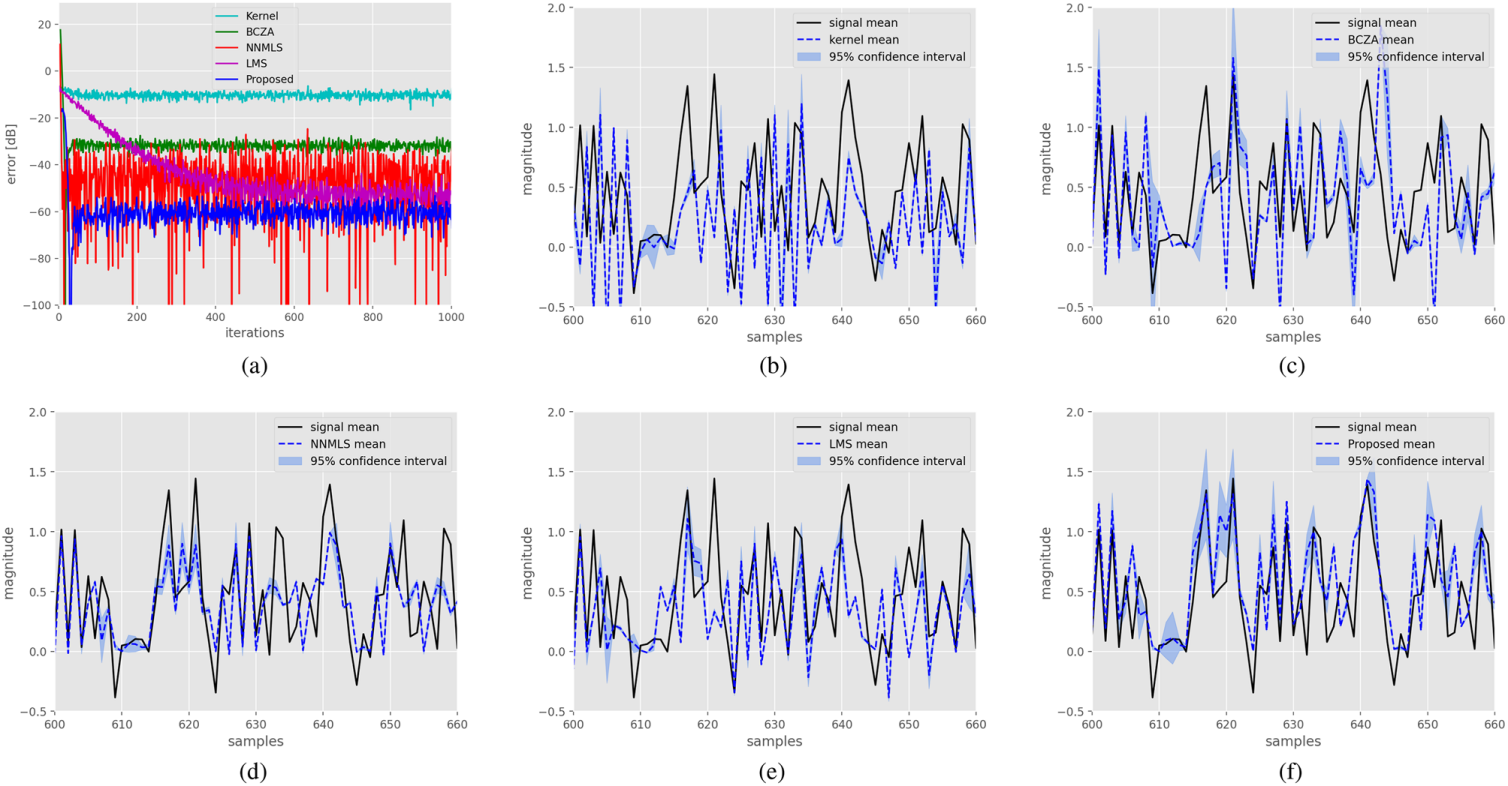
Identification of a system with a spherical manifold structure. Figure (**a**) displays learning curves for the different methods. Figure (**b**) shows 100 realizations of the system response, along with the response of the Kernel filter and $95\%$ confidence intervals. Similarly, Figs. (**c**–**f**) illustrate the performance of the BCZA filter, NNMLS filter, LMS filter, and Proposed filter, respectively, with their corresponding response and confidence intervals in blue shades.

Results for the spherical filter are as follow, the Kernel method achieved a mean squared error (MSE) of $-20.45$ dB, the BCZA method attained $-63.18$ dB, NNMLS obtained $-82.07$ dB, LMS reached $-79.07$ dB, and the proposed method achieved $-117.57$ dB. Figure 3a presents learning curves, demonstrating that the proposed filter achieves the lowest error compared to all other methods, leveraging the inherent structure of the synthetic example. Figure 3b–f exhibit the performance of each filter across 100 realizations of the data. It is also evident from these figures that the proposed method closely tracks the system output. Additionally, it is worth noting that all evaluated methods display high variance in the peaks and flat regions.

**Figure 4:**
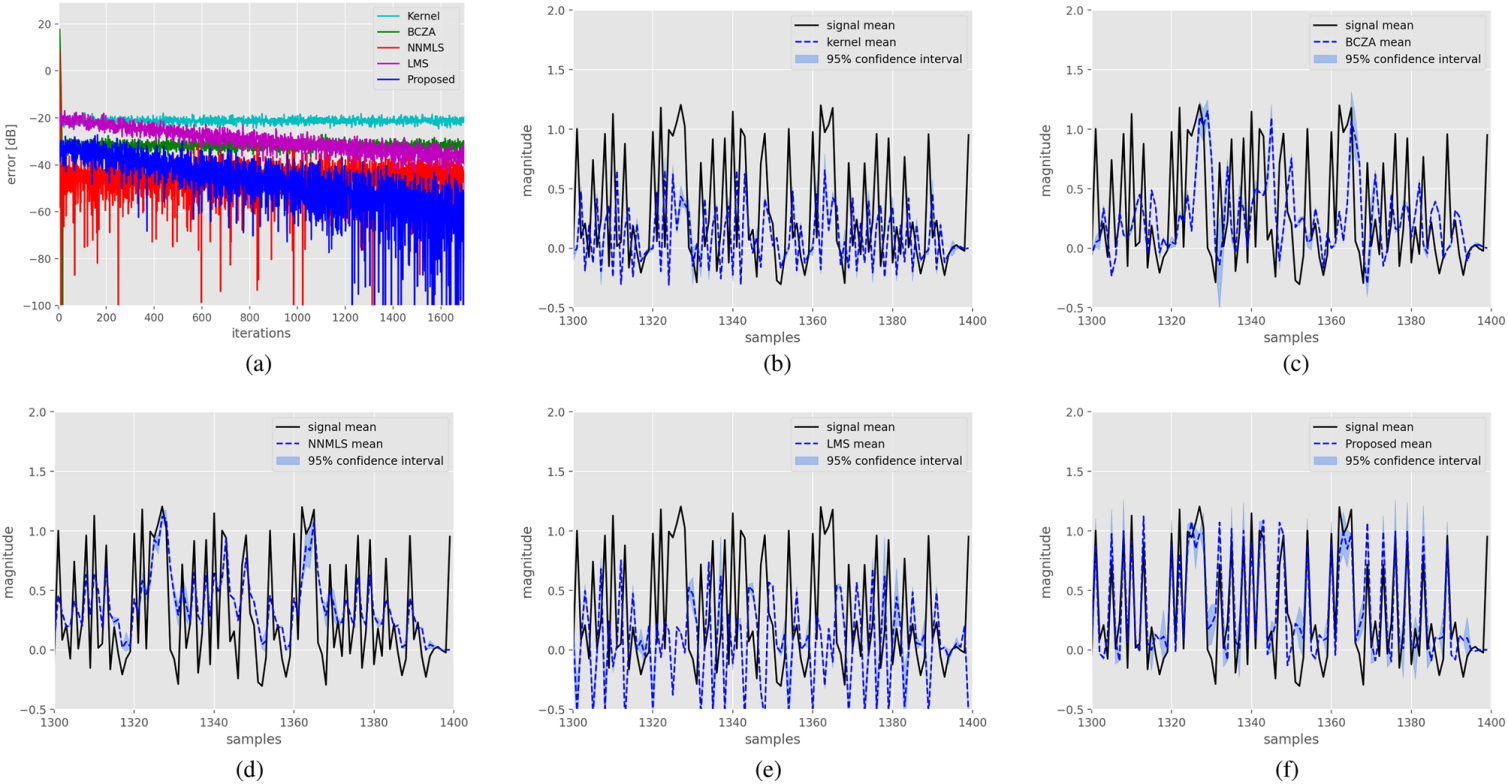
The identification of a system characterized by a hyperbolic manifold structure is presented. Figure (**a**) showcases the learning curves for different methods. Additionally, Fig. (**b**) presents 100 realizations of the system’s response, along with the response of the Kernel filter and corresponding $95\%$ confidence intervals. Similarly, Figs. (**c**–**f**) depict the performance of the BCZA filter, NNMLS filter, LMS filter, and Proposed filter, respectively, along with their response and confidence intervals.

For the system characterized by a hyperbolic structure, the Kernel method achieved a mean squared error (MSE) of $-42.41$ dB, the BCZA method attained $-63.18$ dB, NNMLS obtained $-86.03$ dB, LMS reached $-60.22$ dB, and the proposed method achieved $-89.50$ dB. In Fig. 4a, the learning curves for each method are depicted, with the proposed method demonstrating superior performance compared to the others. Figure 4b–f showcase the system response alongside the response of each method. Notably, the proposed method exhibits superior performance in this case.

**Figure 5:**
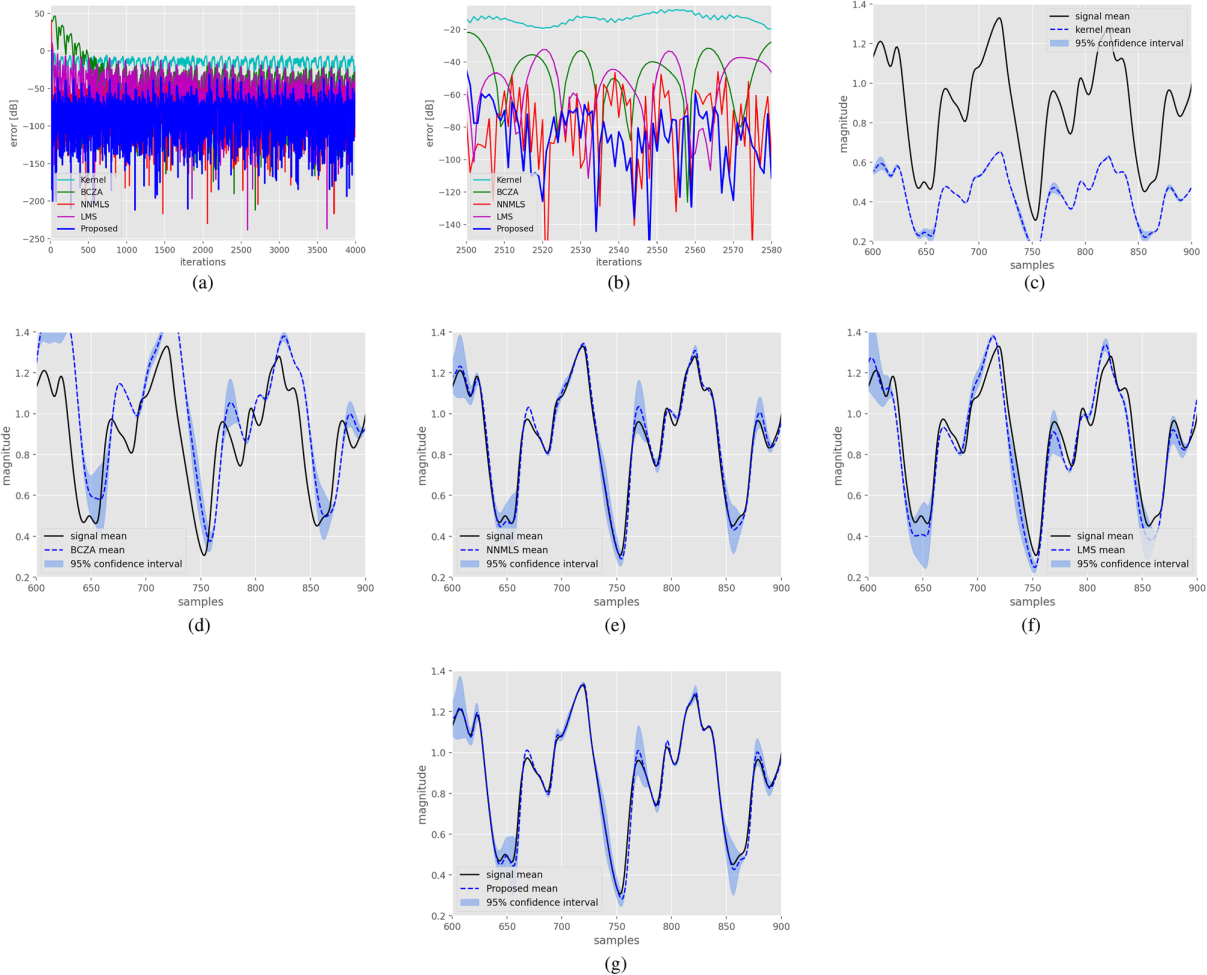
Learning curves for the Mackey-Glass chaotic time series: (**a**) the first 4000 iterations of the series, (**b**) a zoomed-in image of iterations 2500 to 2580 of the methods. Also, $95\%$-confidence intervals are depicted as shadows regions from (**c**–**g**), (**c**) corresponds to the output of kernel method, (**d**) corresponds to the output of BCZA method, (**e**) corresponds to the output of the NNMLS method, (**f**) corresponds to the output of the LMS method, and (**g**) corresponds to the output of the proposed method (MF). Note that it is at the local peaks that the greatest increase in variance occurs.

The next experiment, the Mackey-Glass chaotic time series was used for prediction. Each data $d_k$ was predicted using $x_k=[d_{k-3},d_{k-4},...,d_{k-n-3}]^T$ for filter order, $n=11$. In addition, each $d_k$ was further contaminated with zero mean white Gaussian noise with a 0.001 variance before it was compared with the filter output $w_k^T x_k$. For this experiment, the proposed filter assumed filter coefficients on a hyperbolic manifold. Figure 5a shows the learning curves for each method. Figure 5b is a zoomed-in image of iterations 2500–2580. The proposed method achieved less MSE, $-126.39$ dB on average, followed closely by the NNMLS algorithm, with $-123$ dB, and the LMS with $-60.24$ dB. However, it can be seen in Fig. 5b that both learning curves were separated by more than 10 dB most of the time. Figure 5c–g; illustrate the performance of the different methods to approximate the Mackey–Glass series. The displayed outcomes represent an average of 10 trials. Moreover, the confidence interval for each point on the curve is computed by utilizing 1.96 standard deviations achieved by the algorithms. It is apparent that a most of algorithms display greater variability at the local maxima and minima of the curves in the time series. Conversely, the method proposed exhibits reduced variance and more precisely tracks the signal.

**Figure 6:**
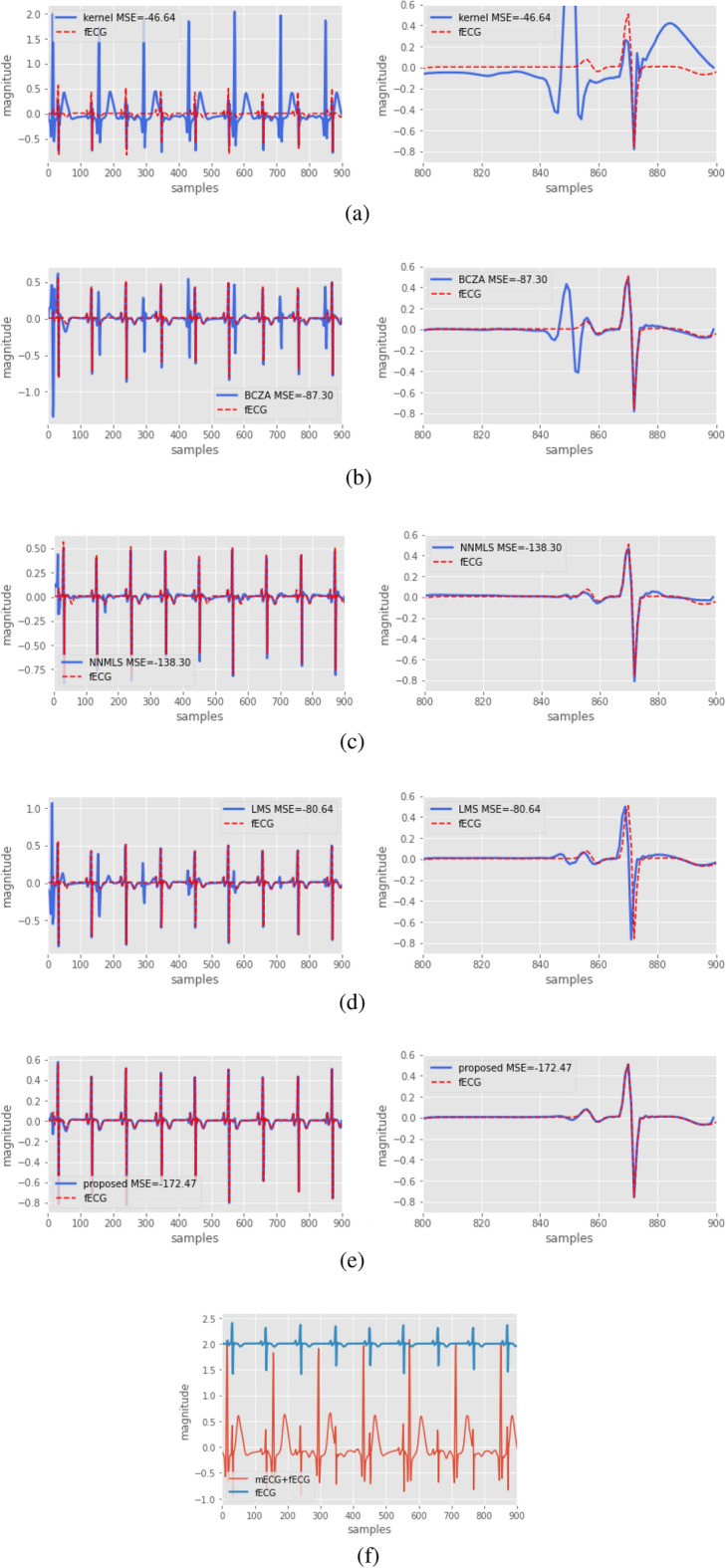
Interference cancellation problem on a simulated fECG. The first column shows the error signal of the filter, and the second column shows a zoom-in of the result for obtained with different methods: (**a**) corresponds to kernel method, (**b**) corresponds to BCZA method, (**c**) corresponds to NNMLS method, (**d**) corresponds to LMS method, and (**e**) corresponds to the proposed method. (**f**) shows, for reference purposes only, the fECG and (fECG $+$ mECG) the signals with a bias of 2.0 bias.

In another experiment, the task was interference cancellation. To this end, simulated fetal electrocardiogram (fECG) data were used, the signal came from the database of simulated mother’s ECG (mECG) and fECG signals^32^ sampled at a rate of 250 samples per second. Figure 6f shows the fECG mounted on a direct current for illustration purposes, and the abdominal ECG signal, which was composed of the mECG and fECG. The signal $d_k$ consisted of the ECG at the mother’s belly (mECG+fECG), the reference signal consisted of the mECG, and the fECG was obtained as the error signal. For this experiment, the proposed method assumed that the filter coefficients are on a hyperbolic manifold, and the filter order for all the algorithms was 21. Figure 6a–e, shown the recovery of the fECG for each method. The right graph shows a zoomed-in image of the response. It can be seen that the proposed method had less MSE and recovered fECG with fewer distortions compared to the other methods.

**Figure 7:**
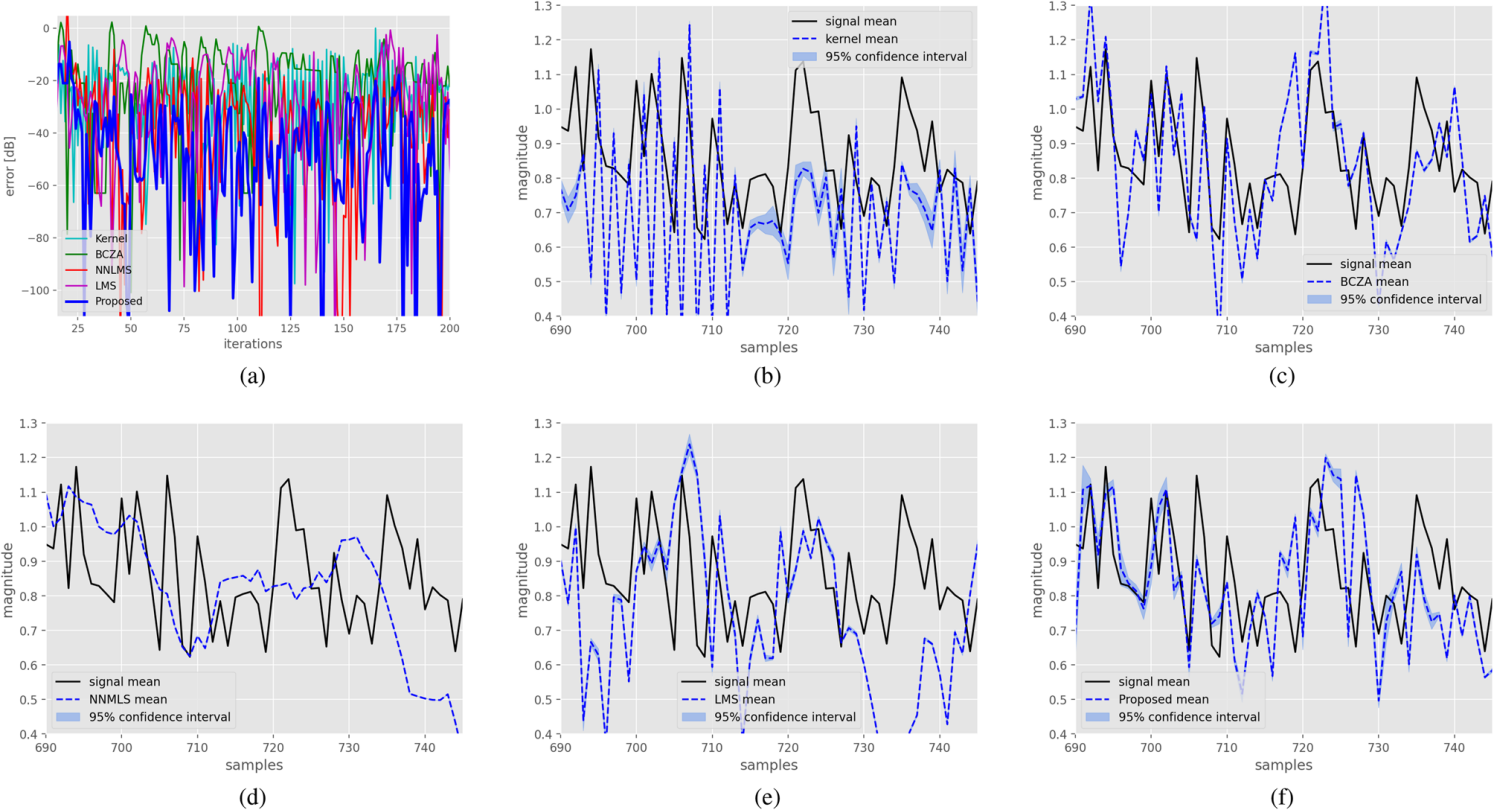
Application of the proposed method to the air heater system data. Figure (**a**) displays the learning curves obtained using the different methods. The responses of the real system for 100 realizations, along with the responses for each filter, are also presented: the kernel filter in (**b**), the BCZA filter in (**c**), the NNMLS filter in (**d**), the LMS filter in (**e**), and the proposed filter in (**f**).

For the next experiment, the task at hand was system identification. The data used were from an air heater to be installed in a production line. The data were acquired at one sample per second. The input signal was a digital signal activating a relay, while the output signal was from a temperature sensor. The proposed filter assumed a hypersphere manifold. The size of all filter was three. The resulting learning curves for the different methods are presented in Fig. 7a. Once again, the proposed method exhibited superior results, achieving a mean squared error (MSE) of $-74.34$ dB. In comparison, the Kernel filter attained $-36.44$ dB, the BCZA method reached $-31.01$ dB, NNLMS with $-63.81$ dB, and the LMS method achieved $-37.72$ dB. Furthermore, in Fig. 7b–f, the system and filter responses for 100 realizations are depicted. It is evident that the proposed method outperforms the others, closely following the signal.

**Figure 8:**
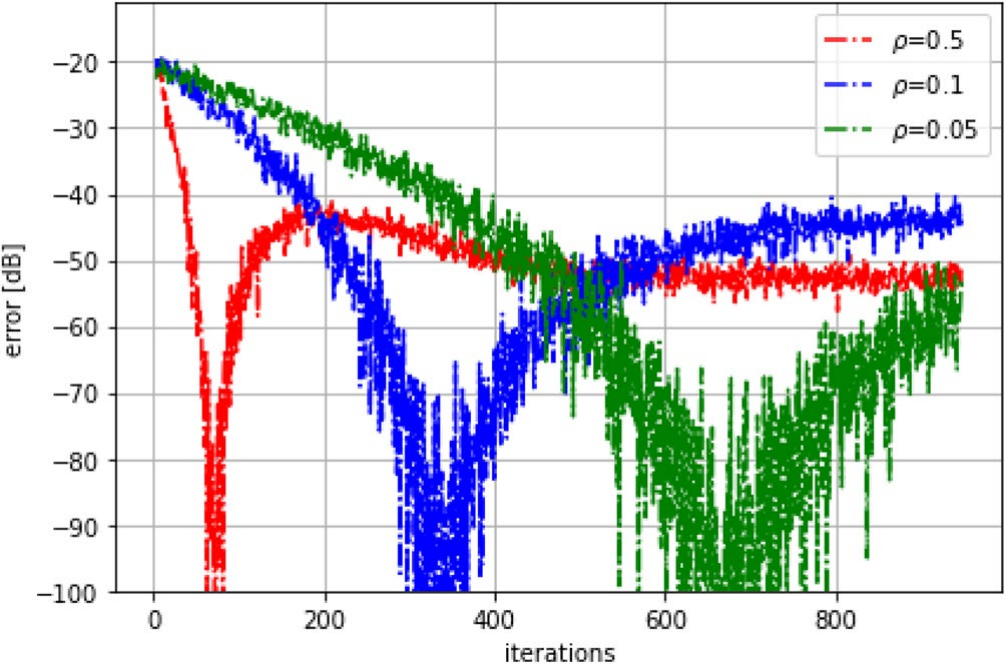
Duration of the output stabilization when the learning rate or $\rho$ parameter is varied for values of 0.5, 0.1, and 0.05. Note that at the lowest learning rate, the proposed algorithm stabilizes with a small error.

Finally, the following experiment aims to assess the method’s sensitivity to the learning rate $\rho$. To accomplish this, a low-pass FIR filter consisting of two coefficients was employed as the system, with an input analogous to that of the preceding experiment. The $\rho$ parameter was varied at multiple values: 0.5, 0.1, and 0.05. As seen in Fig. 8, the duration of the output’s stabilization transient increases as the parameter decreases, and it is also evident that the error diminishes as $\rho$ decreases.

## Conclusions

In this work, an adaptive filter algorithm is proposed. Instead of assuming Euclidean embedding we supposed that the best filter coefficients were embedded in a manifold. We modified the well-known LMS algorithm, considering a manifold with a known structure. We proved the effectiveness of the proposed method for interference cancellation, prediction, and system identification tasks. The results obtained by all the methods showed that the proposed method outperformed all the other methods. The future work should include selecting the right manifold type for the task and using a variable regularizer parameter.

## Data Availability

The data presented in this study are available upon request. Please contact the corresponding author.
